# Combinatorial control of type IVa pili formation by the four polarized regulators MglA, SgmX, FrzS, and SopA

**DOI:** 10.1128/jb.00108-24

**Published:** 2024-10-15

**Authors:** Michel Oklitschek, Luís António Menezes Carreira, Memduha Muratoğlu, Lotte Søgaard-Andersen, Anke Treuner-Lange

**Affiliations:** 1 Department of Ecophysiology, Max Planck Institute for Terrestrial Microbiology, Marburg, Germany; Geisel School of Medicine at Dartmouth, Hanover, New Hampshire, USA

**Keywords:** type IV pili, *Myxococcus xanthus*, MglA, polarity, bacterial motility, SgmX, FrzS, bacterial cell biology

## Abstract

Type IVa pili (T4aP) are widespread and enable bacteria to translocate across surfaces. T4aP engage in cycles of extension, surface adhesion, and retraction, thereby pulling cells forward. Accordingly, the number and localization of T4aP are critical to efficient translocation. Here, we address how T4aP formation is regulated in *Myxococcus xanthus*, which translocates with a well-defined leading and lagging cell pole using T4aP at the leading pole. This localization is orchestrated by the small GTPase MglA and its downstream effector SgmX that both localize at the leading pole and recruit the PilB extension ATPase to the T4aP machinery at this pole. Here, we identify the previously uncharacterized protein SopA and show that it interacts directly with SgmX, localizes at the leading pole, stimulates polar localization of PilB, and is important for T4aP formation. We corroborate that MglA also recruits FrzS to the leading pole, and FrzS stimulates SgmX recruitment. In addition, FrzS and SgmX separately recruit SopA. Precise quantification of T4aP-formation and T4aP-dependent motility in various mutants supports a model whereby the main pathway for stimulating T4aP formation is the MglA/SgmX pathway. FrzS stimulates this pathway by recruiting SgmX and SopA. SopA stimulates the MglA/SgmX pathway by stimulating the function of SgmX, likely by promoting the SgmX-dependent recruitment of PilB to the T4aP machinery. The architecture of the MglA/SgmX/FrzS/SopA protein interaction network for orchestrating T4aP formation allows for combinatorial regulation of T4aP levels at the leading cell pole resulting in discrete levels of T4aP-dependent motility.

**IMPORTANCE:**

Type IVa pili (T4aP) are widespread bacterial cell surface structures with important functions in translocation across surfaces, surface adhesion, biofilm formation, and virulence. T4aP-dependent translocation crucially depends on the number of pili. To address how the number of T4aP is regulated, we focused on *M. xanthus*, which assembles T4aP at the leading cell pole and is a model organism for T4aP biology. Our results support a model whereby the four proteins MglA, SgmX, FrzS, and the newly identified SopA protein establish a highly intricate interaction network for orchestrating T4aP formation at the leading cell pole. This network allows for combinatorial regulation of the number of T4aP resulting in discrete levels of T4aP-dependent motility.

## INTRODUCTION

Bacterial motility is important for the colonization of environmental niches, interactions with host cells, virulence, biofilm formation, and fitness by directing cells toward nutrients and away from toxins and predators (1). For translocation on solid surfaces, bacteria most commonly use type IVa pili (T4aP), long thin filaments that are also important for adhesion to host cells and abiotic surfaces, biofilm formation, virulence, predation, protein secretion, DNA uptake, and surface sensing (2). T4aP undergo cycles of extension, surface adhesion, and retraction (3–5). During these cycles, retractions generate a force of up to 150 pN that is sufficient to pull a cell forward (3, 5, 6). Efficient T4aP-dependent translocation depends on the number and cellular localization of T4aP (7, 8).

The T4aP extension/adhesion/retraction cycles are powered by the highly conserved T4aP machine (T4aPM) (2). In Gram-negative bacteria, this nanomachine is composed of 15 highly conserved proteins and spans from the outer membrane (OM) across the periplasm and inner membrane (IM) to the cytoplasm (9–11) (Fig. S1A). The hexameric PilB and PilT ATPases (12–15) associate with the cytoplasmic base of the core T4aPM in a mutually exclusive fashion to power T4aP extension and retraction, respectively (10). With the exception of PilT, all T4aPM proteins are important for T4aP extension, while PilT is only important for retraction (2). The T4aP is composed of thousands of copies of the major pilin subunit and contains a tip complex composed of minor pilins and the PilY1 adhesin (11, 16–18). During extensions, major pilins are extracted from the IM and inserted at the T4aP base (4, 19, 20); during retractions, this process is inverted, with major pilin subunits being removed from the T4aP base and reinserted into the IM (4, 21). While the highly conserved T4aPM constitutes the basis for the extension/adhesion/retraction cycles, much less conserved regulatory proteins determine where and how many T4aP are formed (7, 18, 22–29). However, their mechanism of action is poorly understood. Here, we address the regulation of T4aP formation in *Myxococcus xanthus*, a predatory soil bacterium with a social lifestyle and a model organism for understanding T4aPM function and regulation.

The rod-shaped *M. xanthus* cells move across surfaces in the direction of their long axis using two motility systems, one for gliding and one for T4aP-dependent motility (30, 31). Motility is important for the social behaviors of *M. xanthus* including predation and formation of swarming colonies in the presence and spore-filled fruiting bodies in the absence of nutrients (30–32). The T4aPM core is present at both cell poles (11, 33–37). However, T4aP only assemble at one pole at a time (38, 39). This localization enables *M. xanthus* cells to move unidirectionally with a piliated leading and a non-piliated lagging cell pole (7, 39) and is essential for efficient translocation across surfaces (7). Consistent with the unipolar T4aP formation, the PilB extension ATPase almost exclusively localizes to the leading cell pole, while the PilT retraction ATPase localizes in a more bipolar asymmetric pattern and with the large cluster at the lagging cell pole (34) (Fig. S1B). In response to signaling by the Frz chemosensory system, *M. xanthus* cells reverse their direction of translocation (40) and after a reversal, T4aP assemble at the new leading pole (39); in parallel, PilB and PilT switch polarity (34) (Fig. S1B).

The activity of the T4aPM in *M. xanthus* is regulated by the polarity module (41–43). The output of this module is generated by the small Ras-like GTPase MglA, which is a nucleotide-dependent molecular switch that is inactive in the GDP-bound and active in the GTP-bound state (44, 45). In its GTP-bound state, MglA localizes to and defines the leading cell pole (44, 45) (Fig. S1C). At this pole, MglA interacts with effectors to stimulate the T4aPM resulting in T4aP formation (7, 46) and is essential for T4aP-dependent motility (47, 48). The remaining five proteins regulate the nucleotide-bound state and localization of MglA by acting as a guanine nucleotide exchange factor in case of the RomR/RomX complex (49) or as a GTPase-activating protein in case of the MglB/RomY complex (44, 45, 50). MglA and the RomR/RomX and MglB/RomY complexes together with the MglC protein interact to bring about their asymmetric polar localization (43, 51) (Fig. S1C). During the Frz-induced reversals, these six proteins switch polarity, thereby enabling the activation of the T4aPM at the new leading cell pole (43–45, 49, 50, 52–54) (Fig. S1C).

At the leading pole, MglA directly interacts with and recruits SgmX, a protein containing 14 tetratricopeptide repeats (TPR) (7, 46), and has also been suggested to interact directly with FrzS (55), which is also important for T4aP-dependent motility (56, 57). FrzS also interacts directly with SgmX and stimulates the recruitment of SgmX to the leading pole (58). SgmX, in turn, brings about PilB localization at the leading pole by an unknown mechanism and is essential for T4aP formation and, consequently, also for T4aP-dependent motility (7). Based on these observations, it has been suggested that SgmX stimulates T4aP formation by enabling PilB interaction with the base of the T4aPM (7).

Here, to increase our understanding of how T4aP formation is regulated in *M. xanthus*, we searched for putative SgmX interaction partners. We identify the previously uncharacterized protein MXAN_0371 (reannotated to MXAN_RS01825 in the NCBI Reference Sequence NC_008095.1; henceforth Stimulation of pili formation protein A, SopA) and demonstrate that SopA interacts directly with SgmX, localizes at the leading pole, stimulates polar PilB localization, and is important but not essential for T4aP-formation and T4aP-dependent motility. We confirm that MglA is important but not essential for FrzS polar localization, and FrzS interacts directly with SgmX, thereby stimulating the polar recruitment of SgmX. In doing so, FrzS indirectly stimulates PilB polar localization, T4aP-formation, and T4aP-dependent motility. Additionally, SgmX and FrzS can separately recruit SopA to the leading pole. Altogether, our data support a model whereby MglA, SgmX, FrzS, and SopA interact to establish a protein interaction network that allows for combinatorial regulation of T4aP formation at the leading cell pole resulting in discrete levels of T4aP-dependent motility.

## RESULTS

### SopA is important for T4aP-dependent motility

We previously identified RomX and RomY using a phylogenomic approach in which we searched for proteins that co-occur with MglA, MglB, and RomR of the polarity module (49, 50). Therefore, to identify proteins that could interact with SgmX, we searched the STRING database (59) for proteins that co-occur with SgmX, resulting in the identification of 10 proteins (Table S1). With the exception of MXAN_5763-_5765 (reannotated to MXAN_RS27935, MXAN_RS27940, and MXAN_RS27945 in the NCBI Reference Sequence NC_008095.1), which are encoded downstream of *sgmX* (7, 28), and deletion of which has no impact on T4aP-dependent motility (60), none of these proteins have previously been analyzed. Three of the remaining seven proteins are predicted to have enzymatic activity and were not considered further. The hypothetical protein SopA (MXAN_0371/MXAN_RS01825) and the TPR domain protein MXAN_6595 (reannotated to MXAN_RS24110) are both highly conserved in Myxococcales with fully sequenced genomes (Fig. S2A), while the PATAN domain [named after the PatA N-terminal domain in which it was first identified (61)] proteins MXAN_3211 (reannotated to MXAN_RS15550) and MXAN_4965 (reannotated to MXAN_RS24110) are less conserved. From here on, we focused on SopA.

**Fig 1:**
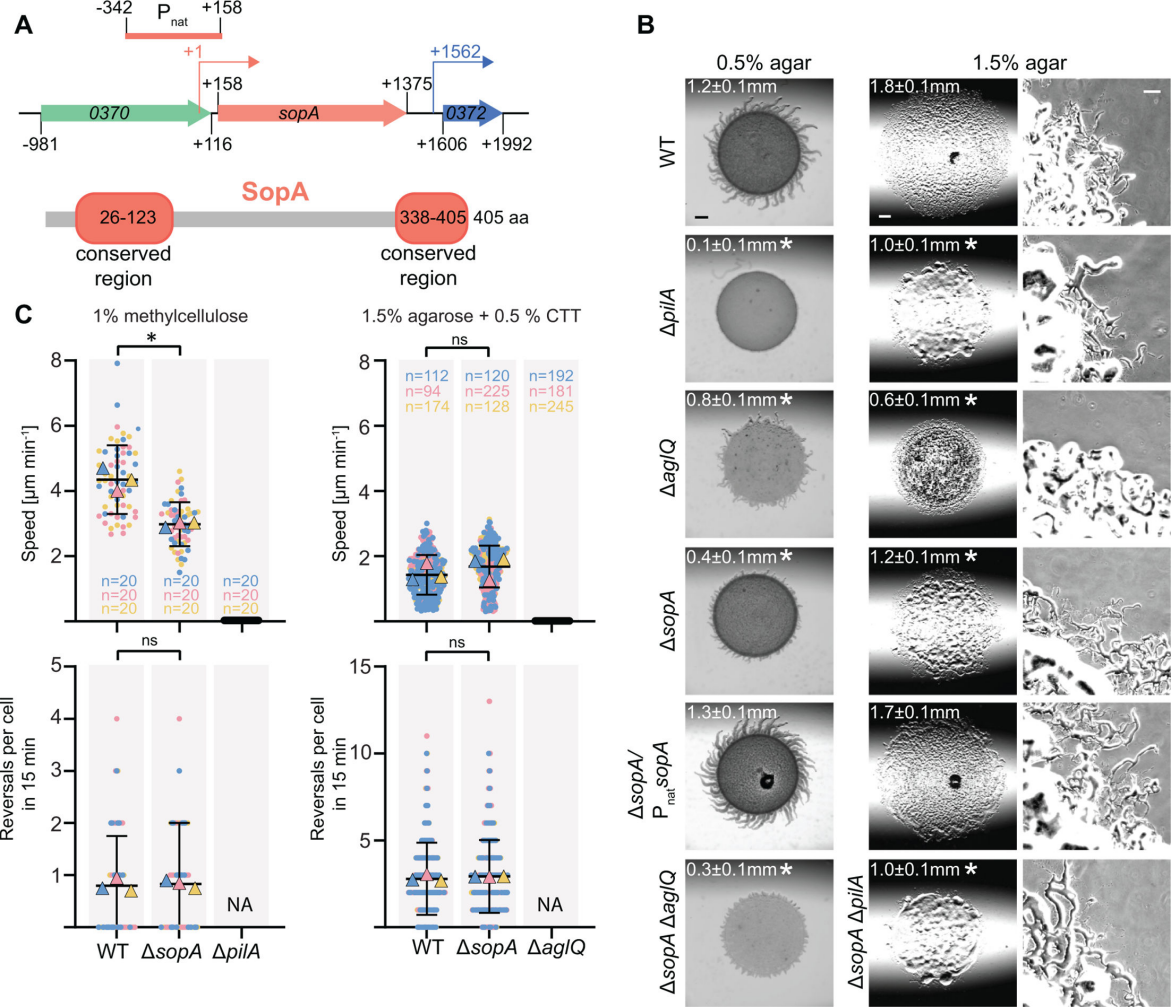
SopA is important for T4aP-dependent motility. (**A**) *sopA* locus and SopA domain architecture. Upper panel, *sopA* locus; numbers in arrows, MXAN locus tags (in the NCBI Reference Sequence NC_008095.1, MXAN_0370, and MXAN_0372 are reannotated as MXAN_RS01820 and MXAN_RS01830, respectively); numbers in black indicate the first and last nucleotide in start and stop codons, respectively, relative to +1, the transcriptional start site of *sopA* (62). Kinked arrows, transcriptional start sites. Light red bar labeled P_nat_ indicates the 500 bp fragment upstream of the *sopA* start codon used for ectopic expression of *sopA*. Lower panel, conserved regions of SopA homologs are indicated in light red using the coordinates of SopA of *M. xanthus*. (**B**) SopA is important for T4aP-dependent motility in population-based assay. T4aP-dependent motility and gliding were analyzed on 0.5% and 1.5% agar supplemented with 0.5% CTT, respectively. Numbers indicate the colony expansion in 24 h as mean ± SD (*n* = 3 biological replicates). *P < 0.05, two-tailed Student’s *t*-test for samples with equal variances. In the complementation strain, *sopA* was expressed from its native promoter from a plasmid integrated in a single copy at the Mx8 *attB* site. Scale bars, 1 mm (left, middle), 100 µm (right). (**C**) SopA is important for T4aP-dependent motility in single-cell-based motility assay. T4aP-dependent motility was measured for cells on a polystyrene surface covered with 1% methylcellulose and gliding on 1.5% agar supplemented with 0.5% CTT. Individual data points from three biological replicates indicated in three different colors and with the number of cells per replicate indicated in the corresponding colors. The mean is shown for each experiment, and the mean for all experiments ± SD is shown in black. *P < 0.05, two-tailed Student’s *t*-test for samples with equal variances, ns, not significant, NA, not applicable because cells are non-motile.

Based on sequence analysis, SopA is a 405 amino acid residue cytoplasmic protein, and homologs were only identified in Myxococcales. SopA homologs share conserved N- and C-terminal regions; however, none of these two regions have sequence similarity to characterized protein domains (Fig. 1A; Fig. S3 and S4A). A high-confidence Alphafold-Multimer structural model of SopA supports that the two conserved regions are folded, while the non-conserved region between these two domains is unstructured (Fig. 1A; Fig. S3 and S4B through D). Finally, a Foldseek search for structural homologs of SopA did not reveal significant structural similarities between the two-folded domains of SopA and other proteins. While the *sopA* locus is conserved in related Myxococcales, none of the genes flanking *sopA* have been implicated in motility (Fig. S2B). Based on RNAseq and cappableseq analyses (62), *sopA* is not encoded in an operon (Fig. 1A).

To characterize a potential function of SopA in motility, we generated a *sopA* in-frame deletion mutant (Δ*sopA*) in the DK1622 wild-type (WT) strain and analyzed the motility characteristics of Δ*sopA* cells in population-based assays. In motility assays on 0.5% agar supplemented with 0.5% casitone broth (CTT), which is most favorable for T4aP-dependent motility (63), WT displayed the long flares at the edge of colonies characteristic of T4aP-dependent motility, while the Δ*pilA* mutant, which lacks the major pilin of T4aP (64) and served as a negative control, generated smooth colony edges without flares (Fig. 1B). The Δ*sopA* mutant formed significantly shorter flares than WT and was significantly reduced in colony expansion (Fig. 1B). This motility defect was complemented by the ectopic expression of *sopA* from its native promoter from a plasmid integrated in a single copy at the Mx8 *attB* site (Fig. 1A and B). Because the Δ*aglQ* mutant, which has a defect in gliding motility due to the lack of a component of the Agl/Glt machinery for gliding (65, 66), also exhibited reduced flare formation on 0.5% agar, we compared flare formation and colony expansion of the Δ*aglQ* mutant and the Δ*sopA*Δ*aglQ* double mutant. The Δ*sopA*Δ*aglQ* double mutant exhibited significantly shorter flares and reduced in colony expansion compared to the Δ*aglQ* mutant (Fig. 1B), documenting that the Δ*sopA* mutation causes a defect in T4aP-dependent motility. On 1.5% agar supplemented with 0.5% CTT, which is most favorable for gliding (63), WT displayed single cells at the edge of the colony, which was not the case for the Δ*aglQ* mutant, which served as a negative control (Fig. 1B). The Δ*sopA* mutant also exhibited single cells at the colony edge but was significantly reduced in colony expansion, and this motility defect was complemented by the ectopic expression of *sopA* (Fig. 1B). Because the Δ*pilA* mutant, while still displaying single cells at the colony edge, also had reduced colony expansion on 1.5% agar, we compared its motility characteristics with those of the Δ*sopA*Δ*pilA* double mutant. These two strains had the same colony expansion, and both had single cells at the colony edge (Fig. 1B). Thus, SopA is not important for gliding motility.

A motility defect in the population-based assay can be caused by a *bona fide* motility defect or by an altered reversal frequency. To distinguish between these two possibilities, we analyzed the single-cell behavior of Δ*sopA* cells. In the single-cell assay for T4aP-dependent motility, cells of the Δ*sopA* mutant displayed a significantly reduced speed compared to WT, while the reversal frequency was unaffected (Fig. 1C). In the single-cell assay for gliding, cells of the Δ*sopA* mutant displayed the same speed and reversal frequency as WT (Fig. 1C).

Based on these motility assays, we conclude that SopA is important but not essential for T4aP-dependent motility and is not important for gliding motility. Moreover, the lack of SopA does not interfere with proper reversals. By comparison, SgmX is essential for T4aP-dependent motility (7, 46).

### SopA is important for T4aP extension

**Fig 2:**
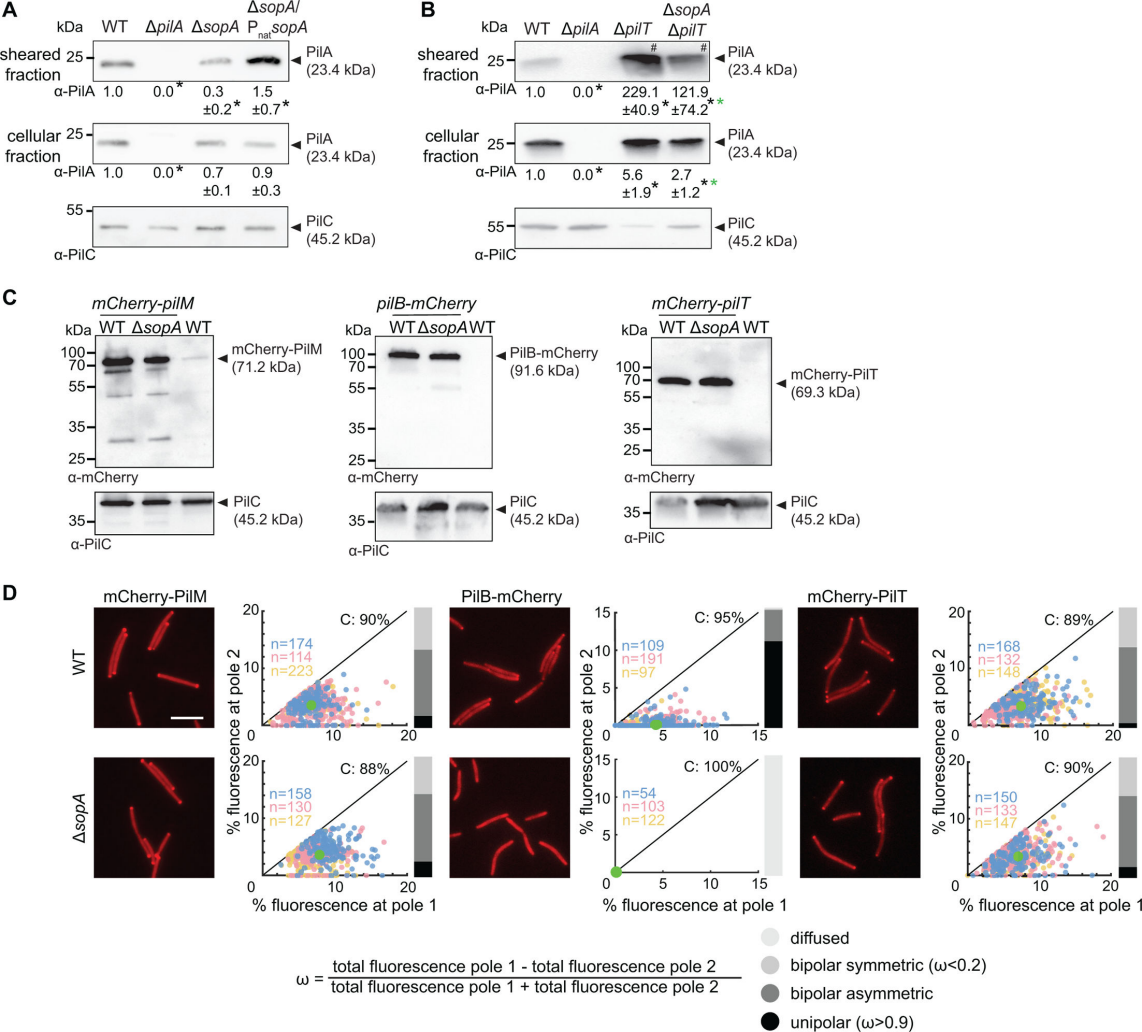
SopA is important for T4aP extension and polar PilB localization. (**A)** SopA is important for T4aP formation. T4aP sheared off from 5 mg cells were separated by SDS-PAGE and probed with α-PilA antibodies (top rows). Middle row, protein from total cell extracts of 10^8^ cells was separated by SDS-PAGE and probed with α-PilA antibodies (middle rows), and after stripping, with α-PilC antibodies as a loading control (bottom rows). Numbers below blots indicate PilA levels as the mean ± SD from four biological replicates relative to WT. *, *P* < 0.05, two-tailed Student’s *t*-test for samples with equal variances. (**B)** SopA is important for T4aP extension. Experiment was done, presented, and analyzed as in A. For better comparison, only 10% of T4aP sheared from the hyper-piliated ∆*pilT* strains (#) were loaded. * (black, green), *P* < 0.05 compared to WT and the ∆*pilT* mutant, respectively. (**C)** Accumulation of mCherry-PilM, PilB-mCherry, and mCherry-PilT in the presence and absence of SopA. Protein from total cell extracts of 10^8^ cells was separated by SDS-PAGE and probed with α-mCherry antibodies (top) and after stripping with α-PilC antibodies as a loading control (bottom). All fusion proteins were synthesized from their native locus. (**D)** Quantification of the polar localization of mCherry-PilM, PilB-mCherry, and mCherry-PilT in the presence and absence of SopA by fluorescence microscopy. Scale bar, 5 µm. In the scatter plots, the percentage of total fluorescence at pole 2 is plotted against the percentage of total fluorescence at pole 1 for all cells with polar cluster(s). Pole 1 is per definition the pole with the highest fluorescence. Individual data points from three independent experiments are shown in three different colors and with the number of cells per replicate indicated in the corresponding colors. Bright green dot, mean fraction of fluorescence at the poles based on all three experiments and including cells with and without clusters. Numbers in the upper right corners, the mean percentage of total cytoplasmic fluorescence based on all three experiments and including cells with and without clusters. Black lines are symmetry lines. For all cells with cluster(s), an asymmetry index, ω, was calculated as indicated; based on ω values, localization patterns were binned into three categories as indicated; diffuse localization was determined when no polar signal was detected. Bar diagrams to the right, the percentage of cells with a polar localization pattern and diffuse localization according to the color code.

To address the mechanism underlying the defect in T4aP-dependent motility in the Δ*sopA* mutant, we examined whether this mutant assembles T4aP using an assay in which T4aP are sheared-off the cell surface followed by quantification of PilA levels by immunoblotting. PilA was still present in the sheared T4aP fraction from the ∆*sopA* mutant but at a significantly reduced level compared to WT, while the total cellular level of PilA was as in WT (Fig. 2A). This defect in T4aP formation was corrected in the complementation strain in which *sopA* was ectopically expressed (Fig. 2A).

Reduced T4aP formation can be caused by impaired T4aP extension or by increased T4aP retraction. To distinguish these two scenarios, we constructed a ∆*sopA*∆*pilT* double mutant, which lacks the PilT retraction ATPase, and determined the piliation level of this strain using the shear-off assay. The non-retracting ∆*pilT* mutant, which assembles a very high level of T4aP (67, 68), as well as the ∆*sopA*∆*pilT* double mutant had significantly higher levels of PilA than WT in the sheared fraction (Fig. 2B; Fig. S5). Importantly, the level of PilA in the sheared fraction of the ∆*sopA*∆*pilT* mutant was significantly lower than in the ∆*pilT* mutant (Fig. 2B; Fig. S5). The ∆*pilT* mutant, in agreement with previous observations (7), and the ∆*sopA*∆*pilT* double mutant both had an increased level of PilA in the cellular fraction (Fig. 2B; Fig. S5). Based on these analyses, we conclude that SopA is important but not essential for T4aP extension. By comparison, SgmX is essential for T4aP extension (7). Of note, the observation that the level of PilA in the sheared fraction in the ∆*sopA*∆*pilT* double mutant is higher than in the ∆*sopA* mutant provides evidence that the ∆*sopA* mutant is able to retract T4aP.

### SopA stimulates polar localization of the PilB extension ATPase

To address how SopA causes a T4aP extension defect, we asked whether a lack of SopA causes a defect in the assembly of the T4aPM. The bipolar assembly of the T4aPM core in *M. xanthus* initiates with the OM secretin PilQ (Fig. S1A) then proceeds in an outside-in pathway culminating with the incorporation of PilM (10, 35, 37). Therefore, we used the bipolar localization of a fully active mCherry-PilM fusion synthesized from the native locus (11) (Fig. S1A) as a proxy for the assembly of the T4aPM core. The fusion protein accumulated at the same level in the WT and the Δ*sopA* mutant (Fig. 2C) and localized similarly in the two strains (Fig. 2D). Also, a fully active mCherry-PilT fusion, which was synthesized from the native locus and accumulated at the same level as native PilT (Fig. S6A), accumulated at the same level in the WT and the Δ*sopA* mutant (Fig. 2C) and localized in the same bipolar asymmetric pattern in the two strains (Fig. 2D). By contrast, the polar localization of a partially active PilB-mCherry fusion, which was synthesized from the native site and accumulated at the same level as native PilB (Fig. S6B), was completely abolished in the absence of SopA (Fig. 2D), while it accumulated independently of SopA (Fig. 2C).

We conclude that SopA is not important for the bipolar assembly of the core T4aPM and the polar localization of PilT; however, SopA is essential for polar localization of the PilB extension ATPase. These observations suggest that the defect in T4aP extension caused by lack of SopA is associated with impaired polar localization of PilB. Importantly, SgmX is also essential for polar localization of PilB but not for polar localization of PilM and PilT (7).

### SopA localizes dynamically to the leading cell pole depending on MglA, SgmX, and FrzS

**Fig 3:**
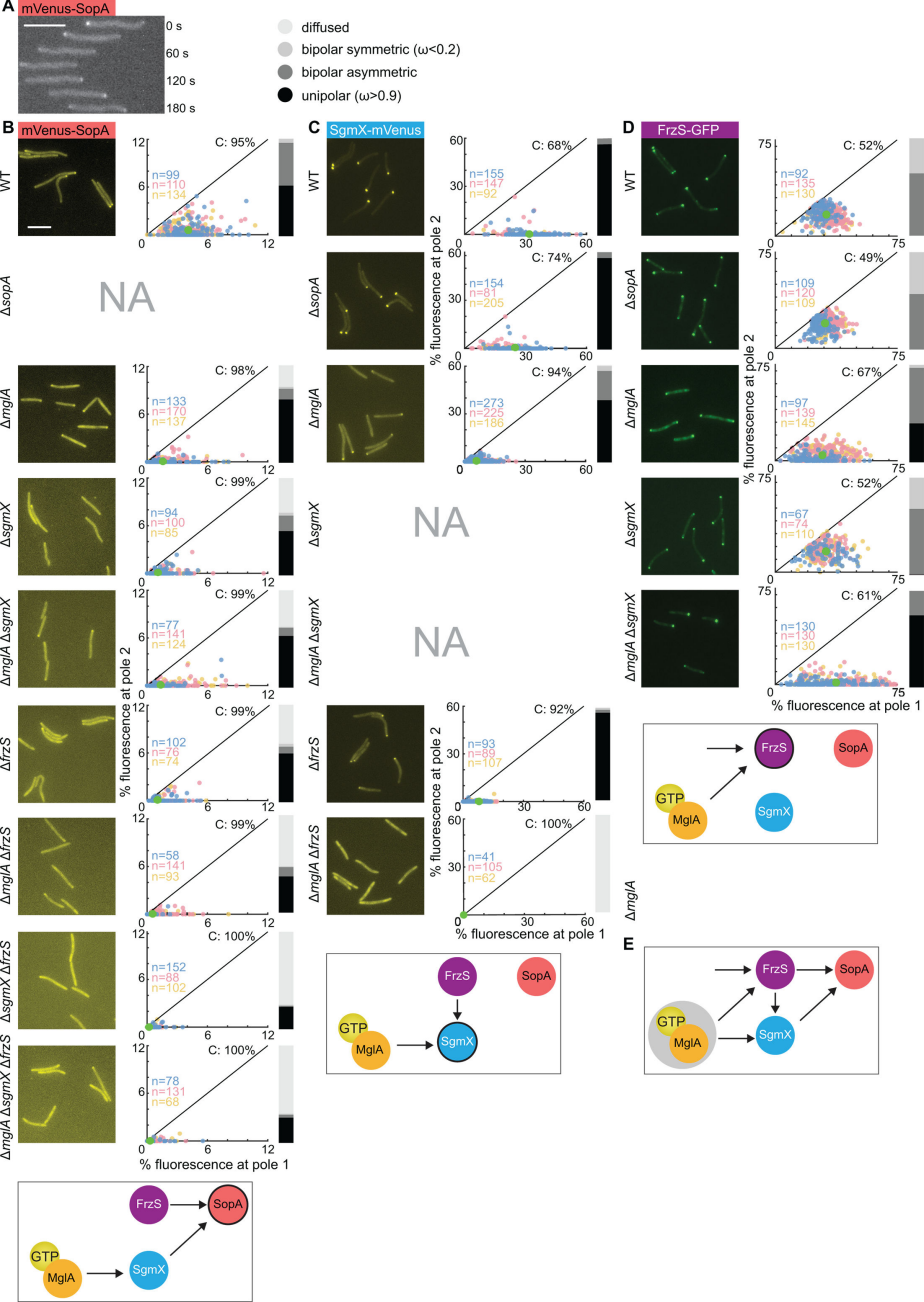
Polar localization of mVenus-SopA, SgmX-mVenus, and FrzS-GFP in the presence and absence of MglA, SopA, SgmX, and/or FrzS. (**A)** mVenus-SopA is dynamically localized with a large cluster at the leading cell pole. Cells were imaged by time-lapse fluorescence microscopy every 30 seconds. Scale bar, 5 µm. (**B–D)** Quantification of the polar localization of mVenus-SopA, SgmX-mVenus, and FrzS-GFP. Experiments were done and are presented as in Fig. 2D. All fusion proteins were synthesized from their native locus. Schematics below each row, summarize effects observed. In the schematics, the protein being analyzed for localization is indicated by black circle.** (E)** Model of protein interaction network for polar localization of MglA, SgmX, FrzS, and SopA. Gray circle surrounding MglA-GTP indicates the polar recruitment of MglA-GTP by the RomR/RomX complex of the polarity module.

To understand the mechanism of SopA in T4aP extension and PilB localization, we asked whether SopA is polarly localized. To this end, we expressed a fully active mVenus-SopA fusion from the native locus (Fig. S7A and B). Using time-lapse fluorescence microscopy and snap-shot image analyses, we observed that mVenus-SopA localized in a unipolar or bipolar asymmetric pattern in all cells and with a large cluster at the leading pole (Fig. 3A and B). During reversals, the polarity of the large cluster was inverted, and after a reversal, it localized at the new leading pole (Fig. 3A).

Next, we asked whether the polar localization of mVenus-SopA depends on MglA and/or SgmX. In the absence of MglA, fewer cells had polar mVenus-SopA clusters, and in cells with cluster(s), these clusters were of lower intensity than in WT (Fig. 3B). In the absence of SgmX, even fewer cells than in the absence of MglA had polar mVenus-SopA clusters, and in cells with cluster(s), these clusters were of lower intensity than in the absence of MglA (Fig. 3B). Because MglA is important for SgmX polar localization, we determined the localization of mVenus-SopA in a Δ*mglA*Δ*sgmX* double mutant and observed that mVenus-SopA largely localized as in the Δ*sgmX* mutant (Fig. 3B). mVenus-SopA accumulated at the same level in all four strains (Fig. S7B). Altogether, these observations suggest a pathway in which MglA recruits SgmX by direct interaction, and then SgmX, in turn, recruits SopA (Fig. 3B).

In the absence of MglA as well as SgmX, more than 50% of cells still had a polar mVenus-SopA signal. We, therefore, hypothesized that an additional protein would be involved in mVenus-SopA polar recruitment. To test this hypothesis, we took a candidate approach and focused on FrzS, which largely co-occurs with SopA (Fig. S2A). In the Δ*frzS* mutant, fewer cells had polar mVenus-SopA clusters, and in cells with cluster(s), these were of lower intensity than in WT (Fig. 3B). In the Δ*mglA*Δ*frzS* double mutant, most cells did not have a polar cluster, and in cells with cluster(s), these were of much lower intensity than in the two strains with a single mutation (Fig. 3B). To test whether SgmX and FrzS have an additive effect on mVenus-SopA polar localization, we generated a Δ*sgmX*Δ*frzS* double mutant. These two mutations had an additive effect on polar mVenus-SopA localization, i.e., most cells did not have polar signal(s), and in the few cells with polar signal(s), these were of very low intensity (Fig. 3B). Finally, in the Δ*mglA*Δ*sgmX*Δ*frzS* triple mutant, mVenus-SopA polar localization was also essentially abolished (Fig. 3B). In all strains, mVenus-SopA accumulated as in WT (Fig. S7B).

We conclude that MglA, SgmX, and FrzS are all important for polar localization of mVenus-SopA. The additive effect of the Δ*sgmX* and Δ*frzS* mutations indicates that SgmX and FrzS provide separate inputs to the polar recruitment of mVenus-SopA. Moreover, our data support that in the SgmX pathway, MglA functions indirectly to recruit SopA by directly recruiting SgmX, which then recruits SopA (Fig. 3B).

### MglA polar localization is independent of SopA

To address whether SopA is important for MglA polar localization, we imaged the localization of MglA-mVenus in WT and Δ*sopA* cells. MglA-mVenus accumulated (Fig. S8A) and localized similarly in WT and the Δ*sopA* mutant (Fig. S8B). Consistently, SopA was neither important for the accumulation nor the polar localization of RomR and MglB, two key proteins of the polarity module (Fig. S1C; Fig. S8A and B). We conclude that SopA acts downstream of the polarity module to stimulate polar localization of PilB and, thereby, T4aP extension and T4aP-dependent motility.

### SgmX polar localization depends on MglA and FrzS but not on SopA

To further understand the interplay between MglA, SgmX, FrzS, and SopA for polar localization, we explored the localization of SgmX. In agreement with previous observations (7, 46), a fully active SgmX-mVenus fusion localized in a highly unipolar pattern in WT and this polar localization was strongly reduced in the Δ*mglA* mutant (Fig. 3C); however, it was not affected in the Δ*sopA* mutant (Fig. 3C). In the absence of FrzS, SgmX-mVenus polar localization was also strongly reduced (Fig. 3C) in agreement with recent observations (58). Moreover, in the Δ*mglA*Δ*frzS* double mutant, SgmX-mVenus polar localization was completely abolished. In all strains, SgmX-mVenus accumulated as in WT (Fig. S7C). These observations suggest that SgmX polar recruitment depends on two pathways: one involves MglA and one involves FrzS (Fig. 3C).

### FrzS polar localization depends on MglA but not on SgmX and SopA

Next, we explored polar FrzS localization. To this end, we used a fully active FrzS-GFP fusion synthesized from the native locus [(69); Fig. S7D]. In agreement with previous observations (53, 55, 69), FrzS-GFP localized in a bipolar asymmetric pattern in WT, and this localization was reduced and shifted to more asymmetric in the absence of MglA (Fig. 3D). FrzS-GFP localization was not affected by the lack of SopA (Fig. 3D). Similarly, FrzS-GFP was not affected by the lack of SgmX (Fig. 3D). Finally, in the Δ*mglA*Δ*sgmX* double mutant, FrzS-GFP localized in the more asymmetric pattern observed in the Δ*mglA* mutant (Fig. 3D). We conclude that MglA is important for polar FrzS-GFP localization, while SgmX and SopA are not (Fig. 3D). We also note that in the Δ*mglA*Δ*sgmX* double mutant, FrzS-GFP formed polar clusters in all cells, documenting that MglA is not the only polar recruitment factor of FrzS. In all tested strains, FrzS-GFP accumulated as in WT (Fig. S7E).

### A highly interconnected protein interaction network establishes the polar localization of MglA, SgmX, FrzS, and SopA

Collectively, the MglA-mVenus, mVenus-SopA, SgmX-mVenus, and FrzS-GFP localization patterns described in Fig. 3B and D; Fig. S8B together with previous findings suggest that multiple interactions between these four proteins establish a highly interconnected network that results in their polar localization (Fig. 3E). In this network, two (MglA and FrzS) of the four proteins can each localize polarly in the absence of the three other relevant proteins. The first protein is MglA, and neither FrzS (53), SgmX (7), nor SopA (Fig. S8B) is important for MglA polar localization, suggesting that MglA is only recruited to the pole *via* the RomR/RomX complex of the polarity module (Fig. 3E). The second protein is FrzS, which can localize polarly independently of MglA, SgmX, and SopA (Fig. 3D). MglA further stimulates FrzS polar localization (Fig. 3D). Downstream of MglA and FrzS, these two proteins can separately recruit SgmX, i.e., MglA can recruit SgmX in the absence of FrzS and *vice versa* (Fig. 3C). Finally, FrzS and SgmX can separately recruit SopA (Fig. 3B). Conversely, SopA neither affects MglA, SgmX, nor FrzS polar localization. In this pathway, the effect of MglA on SopA localization is indirect and depends on the effect of MglA on FrzS and SgmX polar recruitment.

### FrzS is important for T4aP extension and polar PilB localization

**Fig 4:**
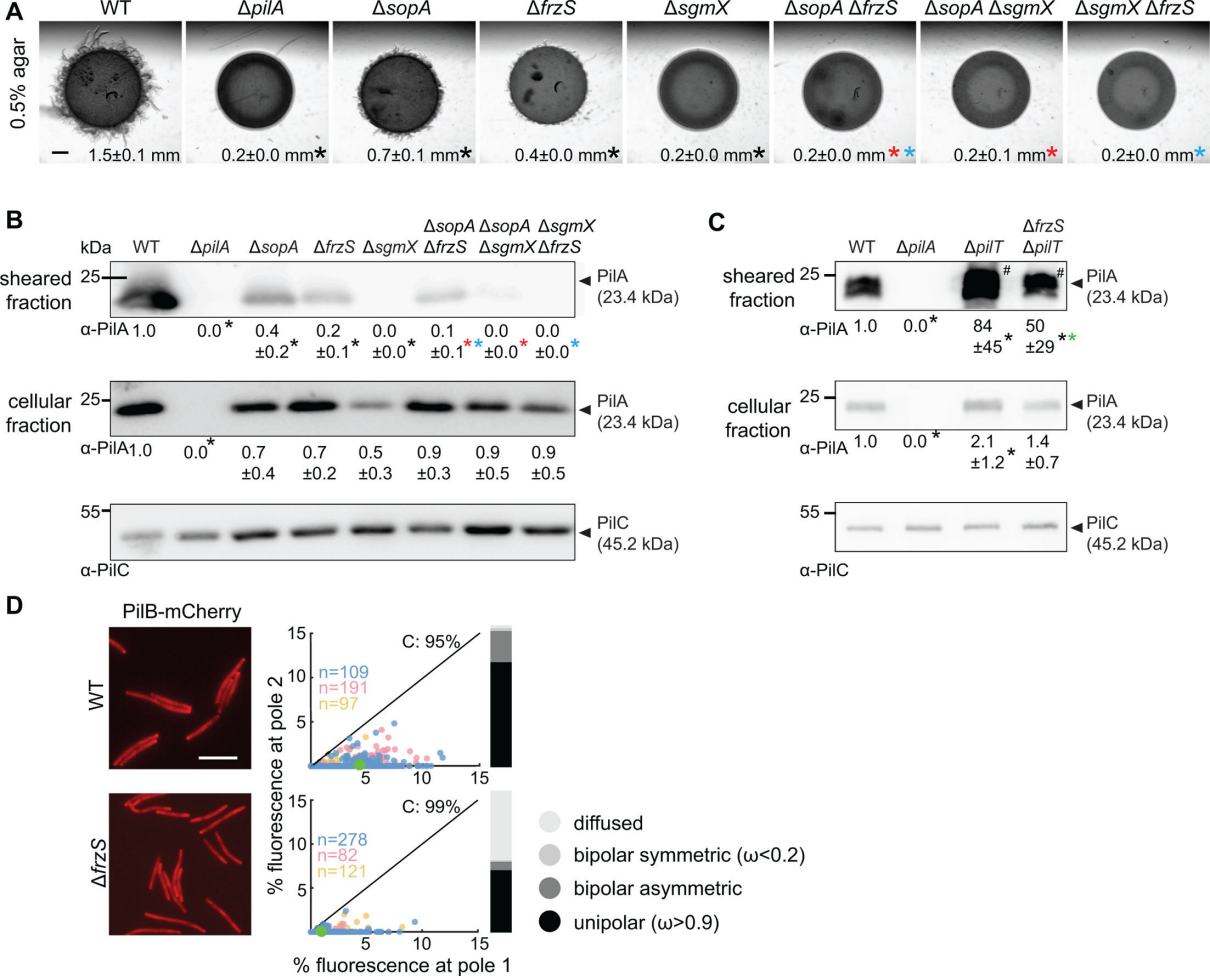
Combinatorial effect of SgmX, FrzS, and SopA on T4aP-dependent motility and T4aP formation.** (A)** Effect of SgmX, FrzS, and/or SopA on T4aP-dependent motility. Cells were incubated on 0.5% agar supplemented with 0.5% CTT. Scale bar, 1 mm. Numbers, colony expansion in millimeter in 24 h as mean ± SD from three biological replicates; * (black, red, and purple) *P* < 0.05, two-tailed Student’s *t*-test for samples with equal variances compared to WT, the Δ*sopA* mutant, and the Δ*frzS* mutant, respectively. (**B and C)** Effect of SgmX, FrzS, and/or SopA on T4aP formation. Experiments were done and data presented as in Fig. 2A and B, except that in B T4aP sheared off from 7.5 mg cells were loaded. * (black, red, blue, and green), *P* < 0.05, two-tailed Student’s *t*-test for samples with equal variances compared to WT, the Δ*sopA* mutant, the Δ*frzS* mutant, and the ∆*pilT* mutant, respectively. (**D)** FrzS is important for polar localization of PilB-mCherry. Experiment was done and data presented as in Fig. 2D.

To examine how the protein interaction network for polar localization of MglA, SgmX, FrzS, and SopA relates to T4aP-formation and T4aP-dependent motility, we first characterized T4aP-dependent motility, T4aP formation, and PilB localization in the Δ*frzS* mutant. In agreement with previous observations (57), the Δ*frzS* mutant had significantly reduced T4aP-dependent motility (Fig. 4A). Moreover, and in agreement with FrzS being important for SgmX and SopA polar localization (Fig. 3E), the Δ*frzS* mutant had significantly reduced PilA in the sheared T4aP fraction (Fig. 4B), and the Δ*frzS*Δ*pilT* double mutant had reduced PilA in the sheared T4aP fraction compared to the Δ*pilT* mutant (Fig. 4C). Furthermore, PilB polar localization was strongly reduced but not abolished in the Δ*frzS* mutant (Fig. 4D). These observations suggest that the defect in T4aP extension caused by lack of FrzS is caused by the reduced polar localization of PilB. Because FrzS is important for polar localization of SgmX and SopA (Fig. 3E), which are, in turn, essential for PilB polar localization, these observations support that the effect of lack of FrzS on T4aP-dependent motility, T4aP formation, and polar localization of PilB is mediated via its effect on SgmX and SopA polar localization.

### The MglA/SgmX/FrzS/SopA interaction network establishes different levels of T4aP-formation and T4aP-dependent motility

Because the analyses of the polar localization of SgmX, FrzS, and SopA in the different genetic backgrounds support an intricate set of interactions between these proteins (Fig. 3E), we hypothesized that these three proteins would have differential effects on T4aP-formation and, thus, T4aP-dependent motility. To test this hypothesis, we compared the defects in T4aP-formation and T4aP-dependent motility in the Δ*sgmX*, Δ*frzS*, and Δ*sopA* mutants and the three double mutants. This comparison revealed that the amount of PilA in the sheared fraction in the six mutants followed a gradient, i.e., the Δ*sopA* mutant had significantly reduced PilA in the sheared fraction, the Δ*frzS* mutant was even more reduced, the Δ*sopA*Δ*frzS* mutant was even more strongly reduced, and PilA in the sheared fraction was undetectable in the Δ*sgmX* mutant and in the two Δ*sopA*Δ*sgmX* and Δ*frzS*Δ*sgmX* double mutants (Fig. 4B). Notably, with the exception of the Δ*sopA*Δ*frzS* mutant, the defects in T4aP formation correlated with the level of T4aP-dependent motility in the different mutants, i.e., it was significantly reduced in the Δ*sopA* mutant, even more strongly reduced in the Δ*frzS* mutant, and abolished in the Δ*sgmX* mutant and the three double mutants (Fig. 4A). The Δ*sopA*Δ*frzS* mutant, which was ~10-fold reduced in the amount of PilA in the sheared fraction compared to WT, did not detectably display T4aP-dependent motility under these test conditions.

### SopA interacts directly with SgmX

**Fig 5:**
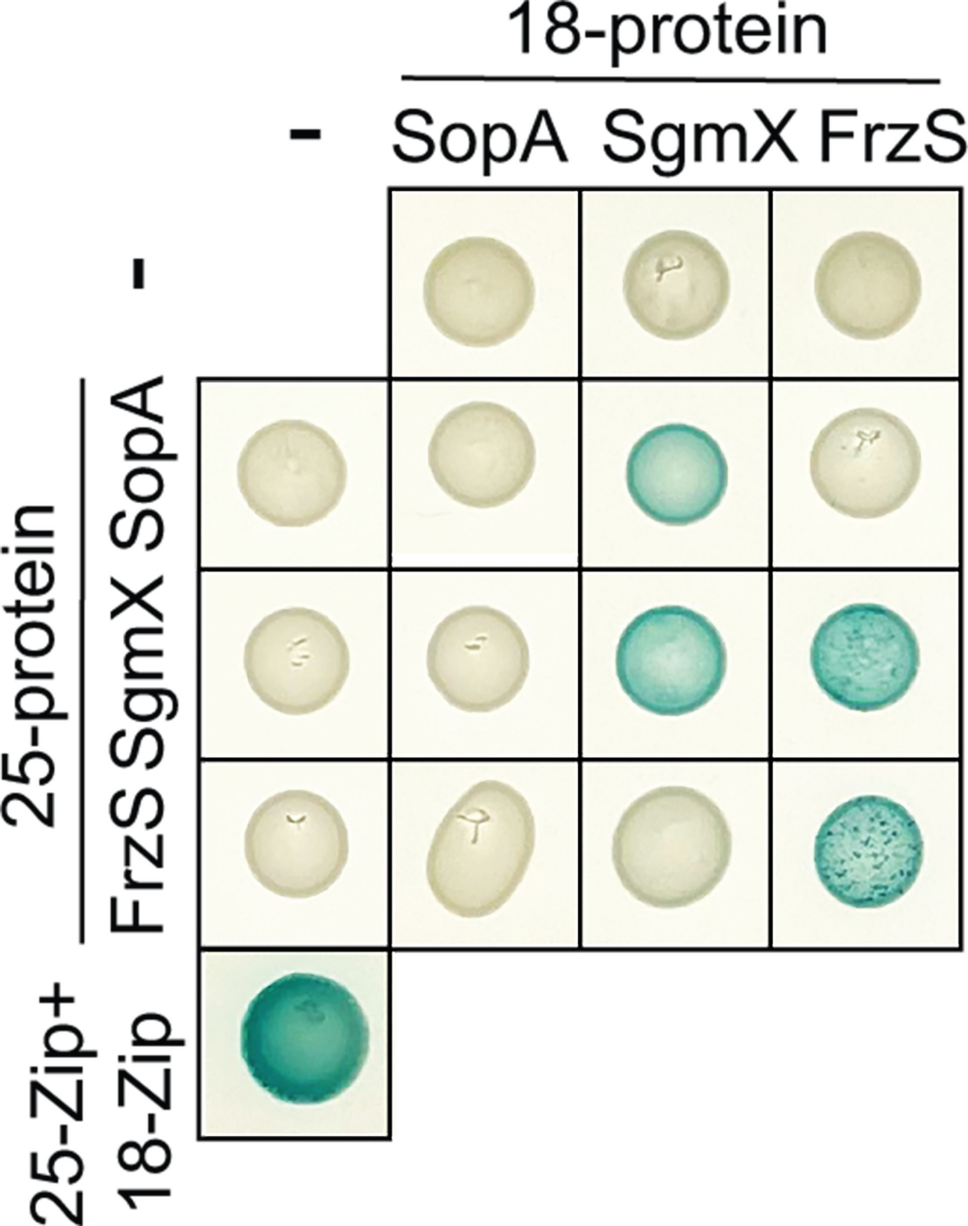
BACTH assay for SgmX, FrzS, and SopA interactions. Full-length SgmX, FrzS, and SopA were fused to the C-terminus of T25 and T18. Lower left corner, T25-Zip + T18 Zip positive control.

SgmX directly interacts with MglA (7, 46) and FrzS (58). Moreover, it has been suggested that MglA interacts directly with FrzS based on *in vivo* pull-down experiments (55). To shed light on whether SopA interacts directly with SgmX and/or FrzS, we used bacterial adenylate cyclase-based two-hybrid (BACTH) analyses (70) with full-length SopA, SgmX, and FrzS proteins. We observed that SgmX as well as FrzS self-interacted (Fig. 5; Fig. S9) in agreement with the observations that purified SgmX and FrzS are both likely dimeric (7, 57). Moreover, we observed interactions between SgmX and FrzS as well as between SgmX and SopA but not between SopA and FrzS (Fig. 5; Fig. S9).

## DISCUSSION

**Fig 6:**
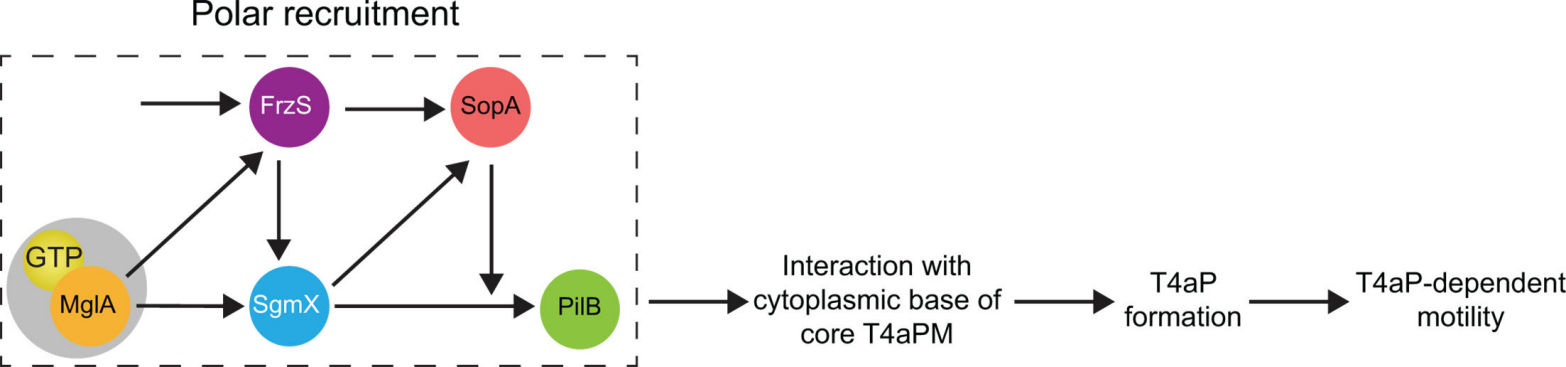
Model of protein interaction network for combinatorial regulation of T4aP-formation and T4aP-dependent motility in *M. xanthus*. The box shown by stippled lines indicates interactions that stimulate polar recruitment of proteins; gray circle surrounding MglA-GTP indicates the polar recruitment of MglA-GTP by the RomR/RomX complex of the polarity module.

In this study, we addressed how T4aP formation is regulated in the rod-shaped cells of *M. xanthus*. Altogether, the detailed quantification of protein localization and T4aP formation supports a model in which the four proteins MglA, SgmX, FrzS, and SopA establish a highly interconnected protein interaction network to regulate T4aP formation (Fig. 6). In this network, the small GTPase MglA is recruited to the leading pole *via* the RomR/RomX complex of the polarity module. MglA and its downstream effector protein SgmX are required and sufficient for the unipolar formation of T4aP and jointly bring about a low level of T4aP formation. By contrast, FrzS and SopA are dispensable for T4aP formation, and our data suggest that these two proteins function to stimulate the MglA/SgmX pathway for T4aP formation. In agreement with previous observations, FrzS is recruited to the leading pole by MglA-dependent and MglA-independent mechanisms. At this pole, FrzS stimulates SgmX polar localization and, thus, T4aP formation. In the case of SopA, it is separately recruited to the leading pole by SgmX and FrzS, where it stimulates the MglA/SgmX pathway for T4aP formation. Because SgmX and SopA are essential for the polar localization of the PilB extension ATPase while FrzS is important, we propose that the output of this pathway is to stimulate PilB interaction with the cytoplasmic base of the core T4aPM (Fig. 6), thereby licensing T4aP formation. Because SopA does not affect the polar localization of MglA, SgmX, and FrzS, we suggest that SopA stimulates the function of SgmX in PilB polar recruitment (Fig. 6).

The detailed quantification of T4aP formation in different mutants supports a model in which the MglA/SgmX/FrzS/SopA interaction network allows for combinatorial regulation of the level of T4aP formation. In this model, the MglA/SgmX/FrzS/SopA interaction network can distinguish different input states that generate output states characterized by discrete levels of T4aP formation. Specifically, (i) in the absence of MglA and SgmX, no T4aP are formed (7), (ii) in the presence of only MglA and SgmX, a low level of T4aP is formed, (iii) in the presence of only MglA, SgmX, and SopA, the level is increased, (iv) in the presence of only MglA, SgmX, and FrzS, an even higher level of T4aP is assembled, and finally, (v) in the presence of all four proteins, the WT level of T4aP formation is accomplished. Thus, this pathway allows the regulation of the number of T4aP by integrating the input from MglA, FrzS, and SopA on the central protein SgmX. Under the conditions of the assay for T4aP-dependent motility, the defects in T4aP formation correlated with the level of T4aP-dependent motility in the different mutants except for the Δ*sopA*Δ*frzS* mutant. This mutant had a ~ 10-fold reduced amount of PilA in the sheared fraction compared to WT and did not display T4aP-dependent motility, suggesting that the number of T4aP in this mutant is too low to enable the pulling of cells across the surface used in the assay for T4aP-dependent motility.

SgmX with its 14 TPRs contains three functional regions (7, 46, 58). The eight N-terminal TPRs mediate the activation of T4aP-dependent motility, the three middle TPRs engage in the interaction to FrzS, and the three C-terminal TPRs in the interaction to MglA (46, 58). FrzS is a pseudo-response regulator with an N-terminal receiver domain, which lacks critical residues for phosphorylation, and a large C-terminal coiled-coil domain (56, 57). The pseudo-receiver domain of FrzS interacts with SgmX (58), while the C-terminal coiled coil is sufficient for polar localization of FrzS (71). Previously, MglA was suggested to interact directly with FrzS (55); however, it is not known how MglA might interact with FrzS. Using a BACTH assay, we observed that SopA interacts directly with SgmX; however, we did not detect an interaction between SopA and FrzS. Based on the dissection of SgmX by Bautista et al. and Mercier et al. (46, 58), we suggest that the eight N-terminal TPRs of SgmX are involved in the polar recruitment of PilB to the T4aPM. PilB interacts directly with PilM and PilC at the cytoplasmic base of the T4aPM [Fig. S1A; (15, 72, 73)]. However, direct interactions between SgmX and PilB and/or PilM have not been detected (7). Interestingly, despite PilB not being polarly localized in the absence of SopA, the Δ*sopA* mutant still makes T4aP, suggesting that the formation of a visible polar PilB cluster may not fully reflect the interaction of PilB with the cytoplasmic base of the core T4aPM. Therefore, important goals for the future will be to determine how SgmX stimulates the interaction of PilB with the cytoplasmic base of the T4aPM and how SopA might further stimulate this interaction.

In other bacteria, the regulation of T4aP formation also centers on the PilB extension ATPase. Specifically, in *Vibrio cholerae* and *Clostridium perfringens*, the second messenger c-di-GMP binds directly to the MshE and PilB2 ATPase, respectively, to stimulate T4aP formation (74–76). In *Xanthomonas axonopodis* pv. citri, c-di-GMP binds to the effector protein FimX, which then interacts with PilZ that, in turn, interacts with PilB, likely to stimulate T4aP formation (23, 77, 78). Similarly, in *Pseudomonas aeruginosa*, the c-di-GMP binding effector proteins FimX stimulate T4aP formation by interacting directly with PilB (25).

The genetic and cell biological analyses demonstrate that the MglA/SgmX/FrzS/SopA network for T4aP formation is able to distinguish different input states with the formation of discrete levels of T4aP. However, the pathway is based on complete loss of function of MglA, SgmX, FrzS, and SopA. Therefore, in the future, it will be interesting to investigate under which physiological conditions these four proteins have altered accumulation and/or localization. In this context, we note that biosynthetic mutants unable to synthesize the secreted polysaccharide exopolysaccharide (EPS) have reduced but not abolished T4aP formation (79). This defect is caused by reduced T4aP extension and not increased retraction (79), but it is not known what causes this extension defect. MglA, SgmX, FrzS, and SopA accumulate at WT levels in an Δ*epsZ* mutant (80) that lacks the phosphoglycosyl transferase EpsZ that initiates EPS biosynthesis (79). In the future, it will be of interest to determine the localization of MglA, SgmX, FrzS, and SopA in EPS biosynthetic mutants.

## MATERIALS AND METHODS

### Cell growth and construction of strains

Strains, plasmids, and primers used in this work are listed in Tables 1 and 2 and Table S2, respectively. All *M. xanthus* strains are derivatives of the DK1622 WT strain (38). *M. xanthus* was grown at 32°C in 1% casitone broth (CTT) (81) or on 1.5% agar supplemented with 1% CTT and kanamycin (50 µg mL^−1^) or oxytetracycline (10 µg mL^−1^) as appropriate. In-frame deletions were generated as described (82). Plasmids were introduced in *M. xanthus* by electroporation and integrated by homologous recombination at the native locus or by site-specific recombination at the Mx8 *attB* site. All in-frame deletions and plasmid integrations were verified by PCR. Plasmids were propagated in *Escherichia coli* TOP10 [F^-^, *mcrA*,Δ(*mrr-hsd*RMS-*mcr*BC), φ80*lac*ZΔM15, Δ*lac*X74, *deoR*, *recA1*, *araD139*, Δ(*ara-leu*)7679, *galU*, *galK*, *rpsL*, *endA1*, and *nu*pG]. *E. coli* was grown in Lysogeny broth (LB) or on plates containing LB supplemented with 1.5% agar at 37°C with added antibiotics when appropriate (83). All DNA fragments generated by PCR were verified by sequencing.

**TABLE 1:** *M. xanthus* strains used in this work

Strain	Genotype	Reference
DK1622	Wild type	(38)
DK10410	Δ*pilA*	(84)
SA5293	Δ*aglQ*	(85)
SA9828	Δ*sopA*	This work
SA9829	Δ*sopA* Δ*pilA*	This work
SA9830	Δ*sopA* Δ*aglQ*	This work
SA9835	Δ*sopA* P_nat_*sopA* (*attB*::pMO28)	This work
DK10409	Δ*pilT*	(68)
SA9859	Δ*sopA* Δ*pilT*	This work
SA7896	*mCherry-pilM*	(11)
SA9300	*pilB-mCherry*	This work
SA9307	*mCherry-pilT*	This work
SA9837	*mCherry-pilM* Δ*sopA*	This work
SA9853	*pilB-mCherry* Δ*sopA*	This work
SA9854	*mCherry-pilT* Δ*sopA*	This work
SA8185	*mglA-mVenus*	(49)
SA3963	*mglB-mCherry*	(54)
SA7507	*romR-mCherry*	(49)
SA9845	*mglA-mVenus* Δ*sopA*	This work
SA9842	*mglB-mCherry* Δ*sopA*	This work
SA9846	*romR-mCherry* Δ*sopA*	This work
SA9848	*mVenus-sopA*	This work
SA9852	*mVenus-sopA* Δ*mglA*	This work
SA9855	*mVenus-sopA* Δ*sgmX*	This work
SA9857	*mVenus-sopA* Δ*frzS*	This work
SA9867	*mVenus-sopA* Δ*frzS* Δ*mglA*	This work
SA9868	*mVenus-sopA* Δ*sgmX* Δ*mglA*	This work
SA9861	*mVenus-sopA* Δ*sgmX* Δ*frzS*	This work
SA9869	*mVenus-sopA* Δ*sgmX* Δ*frzS* Δ*mglA*	This work
SA7164	Δ*sgmX*	(7)
SA7195	*sgmX-mVenus*	(7)
SA9851	*sgmX-mVenus* Δ*sopA*	This work
SA7196	*sgmX-mVenus* Δ*mglA*	(7)
SA9885	*sgmX-mVenus* Δ*frzS*	This work
SA9886	*sgmX-mVenus* Δ*mglA* Δ*frzS*	This work
SA9318	Δ*frzS*	This work
SA9877	Δ*pilT* Δ*frzS*	This work
SA9870	*pilB-mCherry* Δ*frzS*	This work
SA9879	*frzS-gfp*	This work
SA9880	*frzS-gfp* Δ*sopA*	This work
SA9881	*frzS-gfp* Δ*mglA*	This work
SA9882	*frzS-gfp* Δ*sgmX*	This work
SA9883	*frzS-gfp* Δ*sgmX* Δ*mglA*	This work
SA9860	Δ*sopA* Δ*frzS*	This work
SA9856	Δ*sopA* Δ*sgmX*	This work

**TABLE 2:** Plasmids used in this work

Plasmid	Description	Reference
pBJ114	Kan^R^, *galK¸* vector for generating in-frame deletions	(86)
pSWU30	Tet^R^, *attP*	(68)
pKT25	Vector for C-terminal fusion of genes to the T25 fragment of the *Bordetella pertussis* adenylate cyclase gene; kanamycin^R^	Euromedex (BACTH kit)
pKNT25	Vector for N-terminal fusion of genes to the T25 fragment of the *B. pertussis* adenylate cyclase gene; kanamycin^R^	Euromedex (BACTH kit)
pUT18	Vector for N-terminal fusion of genes to the T18 fragment of the *B. pertussis* adenylate cyclase gene; ampicillin^R^	Euromedex (BACTH kit)
pUT18C	Vector for C-terminal fusion of genes to the T18 fragment of the *B. pertussis* adenylate cyclase gene; ampicillin^R^	Euromedex (BACTH kit)
pSL16	pBJ114; for generation of an in-frame deletion of *mglA*	(87)
pLC51	pBJ114; for generation of an in-frame deletion of *sgmX*	(7)
pMAT163	pBJ114; for generation of an in-frame deletion of *pilB*	(11)
pLC20	pBJ114; for integration of *mglA-mVenus* at native locus	(49)
pAP35	pBJ114; for integration of *sgmX-mVenus* at native locus	(7)
pDK145	pBJ114; for integration of *mglB-mCherry* at native locus	(54)
pLC32	pBJ114; for integration of *romR-mCherry* at native locus	(49)
pBJFG	pBJ114; for integration of *frzS-gfp* at native locus	(69)
pLC47	pBJ114; for generation of an in-frame deletion of *sopA*	This work
pMO28	pSWU30; for integration of P_nat_*sopA* at the Mx8 *attB* site	This work
pMO35	pBJ114; for integration of *mVenus-sopA* at native locus	This work
pLC152	pBJ114; for generation of an in-frame deletion of *frzS*	This work
pMEM23	pBJ114; for integration of *pilB-mCherry* at native locus	This work
pMEM33	pBJ114; for integration of *mCherry-pilT* at native locus	This work
pMO41	*sopA* in pKT25	This work
pMO42	*sopA* in pKNT25	This work
pMO43	*sopA* in pUT18	This work
pMO44	*sopA* in pUT18C	This work
pMO45	*frzS* in pKT25	This work
pMO46	*frzS* in pKNT25	This work
pMO47	*frzS* in pUT18	This work
pMO48	*frzS* in pUT18C	This work
pAP29	*sgmX* in pUT18	(60)
pAP30	*sgmX* in pUT18C	(60)
pAP32	*sgmX* in pKT25	(60)
pAP31	*sgmX* in pKNT25	(60)
pKT25-Zip	BACTH control plasmid	Euromedex (BACTH kit)
pUT18C-Zip	BACTH control plasmid	Euromedex (BACTH kit)

### Motility assays and determination of reversal frequency

Population-based motility assays were done as described (63). Briefly, *M. xanthus* cells from exponentially growing cultures were harvested at 4,000 *g* for 10 min at room temperature (RT) and resuspended in 1% CTT to a calculated density of 7 × 10^9^ cells mL^−1^. Five microliter aliquots of cell suspensions were placed on 0.5% agar plates supplemented with 0.5% CTT for T4aP-dependent motility and 1.5% agar plates supplemented with 0.5% CTT for gliding motility and incubated at 32°C. At 24 h, colony edges were visualized using a Leica M205FA stereomicroscope and imaged using a Hamamatsu ORCA-flash V2 Digital CMOS camera (Hamamatsu Photonics) using the LASX software (Leica Microsystems). For higher magnifications of cells at colony edges on 1.5% agar, cells were visualized using a Leica DMi8 inverted microscope and imaged with a Leica DFC9000 GT camera. Single cells were tracked as described (49). Briefly, for T4aP-dependent motility, 5 µL of exponentially growing cultures were placed in a 24-well polystyrene plate (Falcon). After 10 min at RT, cells were covered with 200 µL 1% methylcellulose in MMC buffer {10 mM MOPS [3-(*N*-morpholino) propanesulfonic acid] pH 7.6, 4 mM MgSO_4_, and 2 mM CaCl_2_} and incubated at RT for 30 min. Subsequently, cells were visualized for 15 min at 20-second intervals at RT using a Leica DMi8 inverted microscope with a Leica DFC9000 GT camera and using the LASX software (Leica Microsystems). Individual cells were tracked using Metamorph 7.5 (Molecular Devices) and ImageJ 1.52b (88) and then the speed of individual cells per 20-second interval as well as the number of reversals per cell per 15 min calculated. For gliding, 3 µL of exponentially growing cultures were placed on 1.5% agarose plates supplemented with 0.5% CTT, covered by a cover slide, and incubated at 32°C. After 4–6 h, cells were observed for 15 min at 30-second intervals at RT as described, speed per 30-second interval as well as the number of reversals per 15 min calculated. In both assays, only cells that moved for the entire recording period were included.

### Immunoblot analysis

Immunoblot analysis was done as described (83). Rabbit polyclonal α-PilA (11) (dilution 1:3,000), α-PilC (34) (dilution 1:5,000), α-mCherry (Biovision, dilution 1:15,000), α-PilT (67) (dilution 1:2,000), and α-PilB (67) were used together with horseradish peroxidase-conjugated goat anti-rabbit immunoglobulin G (Sigma) as a secondary antibody (dilution 1:10,000). Monoclonal mouse antibodies were used to detect GFP-tagged proteins (Roche; dilution 1:2,000) together with horseradish peroxidase-conjugated sheep anti-mouse immunoglobulin G (GE Healthcare) as a secondary antibody (dilution 1:2,000). Blots were developed using Luminata Crescendo Western HRP substrate (Millipore) and visualized using a LAS-4000 luminescent image analyzer (Fujifilm). Proteins were separated by SDS-PAGE as described (83).

### T4aP shearing assays

Pili were sheared of *M. xanthus* cells using a protocol based on the procedure of reference (68). Briefly, cells grown on 1% CTT and 1.5% agar plates for 2–3 days were gently scraped off the agar and resuspended in pili resuspension buffer (100 mM Tris-HCl pH 7.6, 150 mM NaCl; 1 mL per 60 mg cells). Cell suspensions were vortexed for 10 min at the highest speed. Cells from a 100 µL aliquot were harvested, the pellet dissolved in 100 µL SDS lysis buffer [10% (vol/vol) glycerol, 50 mM Tris-HCl pH 6.8, 2 mM EDTA, 2% (wt/vol) SDS, 100 mM DTT, and 0.01% bromphenol blue], and immediately denatured at 95°C for 5 min. The remaining suspension was centrifuged for 20 min at 13,000 *g* at 4°C. The supernatant removed and centrifuged twice for 10 min at 13,000 *g* at 4°C to remove cell debris. T4aP in the cell-free supernatant were precipitated by adding 10× pili precipitation buffer (final concentrations: 100 mM MgCl_2_, 500 mM NaCl, and 2% PEG 6000) for at least 2 h at 4°C. The solution was centrifuged for 30 min at 13,000 *g* at 4°C, and the pellet was suspended in SDS lysis buffer (1 µL per mg vortexed cells). T4aP sheared and purified from the same amount of cells were loaded and separated by SDS-PAGE.

### BACTH assays

BACTH assays were performed according to the manufacturer’s protocol (Euromedex). Briefly, plasmids encoding full-length SgmX, FrzS, or SopA fused N-terminally or C-terminally to the T25 or T18 *B. pertussis* adenylate cyclase (CyaA) fragments were transformed into *E. coli* BTH101 [F- *cya*-99 *araD*139 *galE*15 *galK*16 *rpsL1* (Str^r^) *hsdR*2 *mcrA*1 *mcrB*1] alone or in pairs. As a positive control, BTH101 co-transformed with the plasmids pKT25-zip and pUT18C-zip was used. Transformed cells were incubated at 30°C for 24 h. cAMP production by reconstituted CyaA was qualitatively assessed by the formation of blue color as a read out for protein-protein interactions on LB agar supplemented with 40 µg mL^−1^ 5-bromo-4-chloro-3-indolyl-β-d-galactopyranoside and 0.5 mM isopropyl-β-D-thiogalactopyranosid.

### Fluorescence microscopy and image analysis

For fluorescence microscopy, exponentially growing cells were placed on slides containing a thin pad of 1% SeaKem LE agarose (Cambrex) with TPM buffer (10 mM Tris-HCl pH 7.6, 1 mM KH_2_PO_4_ pH 7.6, and 8 mM MgSO_4_) and 0.2% CTT and covered with a coverslip. After 30 min at 32°C, cells were visualized using a temperature-controlled Leica DMi8 inverted microscope and phase contrast and fluorescence images acquired using a Hamamatsu ORCA-flash V2 Digital CMOS camera and the LASX software (Leica Microsystems). For time-lapse recordings, cells were imaged for 15 min using the same conditions. Microscope images were processed with Fiji (89) and cell masks determined using Oufti (90) and manually corrected when necessary. To precisely quantify the localization of fluorescently labeled proteins, we used Matlab R2020a (The MathWorks) in an established analysis pipeline (51) in which the output for each cell is total cellular fluorescence and fluorescence in clusters at each pole. Briefly, cells were segmented, and polar clusters were identified as having an average fluorescence signal of 2 SD above the mean cytoplasmic fluorescence and a size of three or more pixels. Pole 1 was assigned to the pole with the highest fluorescence. For each cell with polar clusters, an asymmetry index (ω) was calculated as:


$$\omega =\frac{totalfluorescenceatpole1-totalfluorescenceatpole2}{totalfluorescenceatpole1+totalfluorescenceatpole2}$$

The localization patterns were binned from the ω values as follows: unipolar (ω > 0.9), bipolar asymmetric (0.9 ≥ ω ≥ 0.2), and bipolar symmetric (ω < 0.2). Diffuse localization was determined when no polar signal was detected. Data points for individual cells were plotted in scatterplots. For calculating mean fraction of polar and cytoplasmic fluorescence, cells with and without clusters were included.

### AlphaFold-Multimer model building

The SopA structural model was generated using AlphaFold-Multimer (Version 2.3.1) via ColabFold (91–93). Five models were generated and ranked based on combined pLDDT (predicted Local Distance Difference Test) and pAE (predicted Alignment Error) values (91). The pLDDT and pAE graphs of the five models were visualized using a custom Matlab R2020a (The MathWorks) script (94). Per residue model accuracy was estimated based on pLDDT values (>90, high accuracy; 70–90, generally good accuracy; 50–70, low accuracy; <50, should not be interpreted) (92). Relative domain positions were validated by pAE. The pAE graphs indicate the expected position error at residue X if the predicted and true structures were aligned on residue Y; the lower the pAE value, the higher the accuracy of the relative position of residue pairs and, consequently, the relative position of domains/subunits/proteins (92). PyMOL version 2.4.1 (Schrödinger LLC) was used to analyze and visualize the models. Foldseek (95) was used to search for structural homologs of SopA.

### Bioinformatics

The search of the STRING database (59) for proteins that co-occur with SgmX was conducted in October 2016. Sequence alignments were generated using ClustalOmega (96) with default parameters, and alignments were visualized with Jalview (97). Protein domains were identified using Interpro (98). Orthologs were identified using the KEGG SSDB database (99). Percent similarity/identity between proteins was calculated using EMBOSS Needle software (pairwise sequence alignment) (100). Phylogenetic trees were prepared in MEGA7 (101) using the Neighbor-Joining method.

### Statistics

Statistics were performed using a two-tailed Student’s *t*-test for samples with equal variances.

## Data Availability

The authors declare that all data supporting this study are available within the article and its Supplementary materials. All materials used in the study are available from the corresponding author.

## SUPPLEMENTAL MATERIAL

Supplemental material jb.00108-24-s0001.docx Fig. S1 to S9; Tables S1 and S2.

ASM does not own the copyrights to Supplemental Material that may be linked to, or accessed through, an article. The authors have granted ASM a non-exclusive, world-wide license to publish the Supplemental Material files. Please contact the corresponding author directly for reuse.
